# Supplementary material for: Separated or joint models of repeated multivariate data to estimate individuals’ disease trajectories with application to scleroderma
Source: PLoS One. 2025 Apr 21;20(4):e0320414. doi: 10.1371/journal.pone.0320414 (PMC12011310; doi:10.1371/journal.pone.0320414)
Supplement: Supporting materials — includes four sections: S1 Efficiency of fixed effect estimates and seemingly unrelated regressions S2 Mean squared error and bias-variance decomposition S3 Mean squared error and bias-variance decomposition of random effect estimates with known population parameters S4 Efficiency gains for the random effects in the case of drop-out missing pattern. (PDF) [file pone.0320414.s001.pdf]

Separated or joint models of repeated multivariate data to estimate individuals' disease trajectories with application to scleroderma

Supporting Materials

Ji Soo Kim[*1,2], Ami A. Shah[1], Laura K. Hummers[1], Scott L. Zeger[2]

1 Division of Rheumatology, Department of Medicine, Johns Hopkins University School of Medicine, Baltimore, Maryland, United States of America
2 Department of Biostatistics, Johns Hopkins Bloomberg School of Public Health, Baltimore, Maryland, United States of America

* Corresponding author
E-mail: jkim478@jhu.edu

1 Efficiency of fixed effect estimates and seemingly unrelated regressions

Zellner (1962) [1] introduces situations where the separated models yield fixed effects estimators as efficient as those estimated in a single multivariate outcome model. We present Zellner's results under his SUR framework and investigate whether or not the results are applicable to the estimates of fixed effects and random effects in our model.

We start with a simplified version of our model. Suppose each of our K measurements includes m individuals responses collected at times $t = 1, ...T$ and $n = m \times T$. The seemingly unrelated regression equations are defined as: $Y_k = X_k\beta_k + \epsilon_k$, $k = 1, ..., K$ where Y_k and ϵ_k are $(n \times 1)$, X_k is $(n \times p)$ and β_k is $(p \times 1)$. The covariances in the errors or disturbance terms ϵ_k are defined across the five measures by $Cov(\epsilon_{kt}, \epsilon_{lt}) = \sigma_{kl} I_n$.

It is possible and often convenient to estimate the regression coefficients for each outcome separately. This is the reason that the equations are called seemingly unrelated regression equations. However, the equations are connected to one another by the correlation among the error terms across the equations.

We can also write this model as one large linear model:

$$Y = \begin{pmatrix} Y_1 \\ Y_2 \\ Y_3 \\ \vdots \\ Y_K \end{pmatrix} = \begin{pmatrix} X_1 & 0 & 0 & \cdots & 0 \\ 0 & X_2 & 0 & \cdots & 0 \\ 0 & 0 & X_3 & \cdots & 0 \\ 0 & 0 & 0 & \ddots & \vdots \\ 0 & 0 & 0 & \cdots & X_K \end{pmatrix} \begin{pmatrix} \beta_1 \\ \beta_2 \\ \beta_3 \\ \vdots \\ \beta_K \end{pmatrix} + \begin{pmatrix} \epsilon_1 \\ \epsilon_2 \\ \epsilon_3 \\ \vdots \\ \epsilon_K \end{pmatrix} = X\beta + \epsilon.$$

With the following covariance matrix for the error term ϵ,

$$V(\epsilon) = \begin{pmatrix} \sigma_{11}I_n & \sigma_{12}I_n & \cdots & \sigma_{1K}I_n \\ \sigma_{21}I_n & \sigma_{22}I_n & \cdots & \sigma_{2K}I_n \\ \vdots & \vdots & & \vdots \\ \sigma_{K1}I_n & \sigma_{K2}I_n & \cdots & \sigma_{KK}I_n \end{pmatrix} = \begin{pmatrix} \sigma_{11} & \sigma_{12} & \cdots & \sigma_{1K} \\ \sigma_{21} & \sigma_{22} & \cdots & \sigma_{2K} \\ \vdots & \vdots & & \vdots \\ \sigma_{K1} & \sigma_{K2} & \cdots & \sigma_{KK} \end{pmatrix} \bigotimes I_n$$
$$= \Sigma \bigotimes I_n$$

σ_{kl} is the covariance between the error term in the equation for measure k and that in the equation for measure l, and σ_{kk} is the variance of the error term in the equation for measure k. The above formulation

assumes that σ_{kk} and σ_{kl} are constant for all observations within respective measures, that there is no correlation between errors of observations collected at different times implying no serial correlation of error terms. More importantly, we are disregarding all within-individual correlation captured by introducing random effects. We will later accommodate our mixed effects model assumptions and modify the results accordingly, but we first demonstrate Zellner's results using our simpler model.

The best linear unbiased estimator for β is the GLS estimates $\hat{\beta} = (X^T V^{-1} X)^{-1} X^T V^{-1} Y$ where

$$V^{-1}(\epsilon) = \begin{pmatrix} \sigma^{11} I_n & \sigma^{12} I_n & \cdots & \sigma^{1K} I_n \\ \sigma^{21} I_n & \sigma^{22} I_n & \cdots & \sigma^{2K} I_n \\ \vdots & \vdots & & \vdots \\ \sigma^{K1} I_n & \sigma^{K2} I_n & \cdots & \sigma^{KK} I_n \end{pmatrix} = \begin{pmatrix} \sigma^{11} & \sigma^{12} & \cdots & \sigma^{1K} \\ \sigma^{21} & \sigma^{22} & \cdots & \sigma^{2K} \\ \vdots & \vdots & & \vdots \\ \sigma^{K1} & \sigma^{K2} & \cdots & \sigma^{KK} \end{pmatrix} \bigotimes I_n$$
$$= \Sigma^{-1} \bigotimes I_n$$

The first of the two conditions under which the equation-by-equation model yields estimators as efficient as $\hat{\beta}$ is when the error terms have a diagonal covariance matrix such that $\sigma_{kl} = \sigma_{lk} = 0$. When we force all covariance terms across measures to be 0, the GLS estimator reduces to K single-equation least-squares estimators. The less obvious condition is $X_1 = X_2 = \cdots = X_K$.

If $X_k = X_1$ for all $k = 1, \ldots, K$,

$$\hat{\beta} = \{X^T(\Sigma^{-1} \bigotimes I_n)X\}^{-1} X^T(\Sigma^{-1} \bigotimes I_n)Y = (\Sigma^{-1} \bigotimes X_1^T X_1)^{-1}(\Sigma^{-1} \bigotimes X_1^T)Y$$
$$= (\Sigma \bigotimes (X_1^T X_1)^{-1})(\Sigma^{-1} \bigotimes X_1^T)Y = (\Sigma \bigotimes (X_1^T X_1)^{-1})(\Sigma^{-1} \bigotimes X_1^T)Y$$
$$= (\Sigma\Sigma^{-1}) \bigotimes ((X_1^T X_1)^{-1} X_1^T)Y = I_m \bigotimes ((X_1^T X_1)^{-1} X_1^T)Y$$
$$= \begin{pmatrix} (X_1^T X_1)^{-1} X_1^T Y_1 \\ \vdots \\ (X_1^T X_1)^{-1} X_1^T Y_K \end{pmatrix}$$

Hence, $\hat{\beta}$ reduces to a vector of single-equation estimators even with correlated error terms. In practice, this implies that when all measures have the same design matrix (i.e. data for all K measures measured at the same time and function of time is the only explanatory variable for each measure), we obtain the same population estimates from the equation-by-equation model and the joint model. We now investigate if these two results also hold under the assumptions of the combined model.

The main difference between the SUR model and the combined model is that patient-specific random effects are introduced. The fixed effect estimates of the combined model is $\hat{\beta}_C = (X^T W_C X)^{-1} X^T W_C Y$ where $W_C^{-1} = V_C = Z\Gamma_C Z^T + \Sigma_C$. We define

$$b_{Ci} = \begin{pmatrix} b_{i1} \\ b_{i2} \\ \vdots \\ b_{iK} \end{pmatrix} \overset{ind}{\sim} N\left(0, \begin{pmatrix} D_{11} & D_{12} & \cdots & D_{1K} \\ D_{21} & D_{22} & \cdots & D_{2K} \\ \vdots & \vdots & & \vdots \\ D_{K1} & D_{K2} & \cdots & D_{KK} \end{pmatrix}\right)$$

and

$$\Gamma_C = \begin{pmatrix} D_{11} \bigotimes I_m & D_{12} \bigotimes I_m & \cdots & D_{1K} \bigotimes I_m \\ D_{21} \bigotimes I_m & D_{22} \bigotimes I_m & \cdots & D_{2K} \bigotimes I_m \\ \vdots & \vdots & & \vdots \\ D_{K1} \bigotimes I_m & D_{K2} \bigotimes I_m & \cdots & D_{KK} \bigotimes I_m \end{pmatrix},$$

$$\Sigma_C = \begin{pmatrix} \sigma_{11} & \sigma_{12} & \cdots & \sigma_{1K} \\ \sigma_{21} & \sigma_{22} & \cdots & \sigma_{2K} \\ \vdots & \vdots & & \vdots \\ \sigma_{K1} & \sigma_{K2} & \cdots & \sigma_{KK} \end{pmatrix} \bigotimes I_n$$

Let Z_{ki} be the random effects design matrix of kth measure of ith person, $Z_k = \bigoplus_{i=1}^{m} Z_{ki}$ the design matrix for measure k, and $Z = \bigoplus_{i=1}^{K} Z_k$.

Then, V_C is

$$\begin{pmatrix} Z_1 D_{11} \bigotimes I_m Z_1^T + \sigma_{11} I_n & Z_1 D_{12} \bigotimes I_m Z_2^T + \sigma_{12} I_n & \cdots & Z_1 D_{1K} \bigotimes I_m Z_K^T + \sigma_{1K} I_n \\ Z_2 D_{21} \bigotimes I_m Z_1^T + \sigma_{21} I_n & Z_2 D_{22} \bigotimes I_m Z_2^T + \sigma_{22} I_n & \cdots & Z_2 D_{2K} \bigotimes I_m Z_K^T + \sigma_{2K} I_n \\ \vdots & \vdots & & \vdots \\ Z_K D_{K1} \bigotimes I_m Z_1^T + \sigma_{K1} I_n & Z_K D_{K2} \bigotimes I_m Z_2^T + \sigma_{K2} I_n & \cdots & Z_K D_{KK} \bigotimes I_m Z_K^T + \sigma_{KK} I_n \end{pmatrix}$$

The off-diagonal block matrices of V_C explaining the covariance of the observations across measures in time become 0 when we have a diagonal covariance matrix for error terms ($\sigma_{kl} = \sigma_{lk} = 0$) and additionally for random effects ($D_{kl} = D_{lk} = 0$). Then, $V_C = V_S$ and $\hat{\beta}_C = \hat{\beta}_S$, and $\hat{\beta_S}$ becomes BLUE.

The remaining question is whether $\hat{\beta}_C = \hat{\beta}_S$ in the case of $X_1 = \cdots = X_K$. As shown above, Zellner's result largely depends on the covariance of the error terms adopted in the SUR model, where it can be written as $\Sigma \bigotimes I_n$. But the form of V_C makes it clear that it cannot be decomposed into a Kronecker product of a matrix with scalar variance terms and the identity matrix for most cases.

Let's now consider a set of estimating equations for subject i, where $Z_i = \bigoplus_{k=1}^{K} Z_{ik}$ is a sub-matrix that consists of columns of $X_i = \bigoplus_{k=1}^{K} X_{ik}$ for measures $k = 1, ..., K$. Then, $Y_i = X_i\beta + Z_i b_i + \epsilon_i$ reduces to $Y_i = X_i(\beta + b_i) + \epsilon_i = X_i\beta_i + \epsilon_i$. If $X_{i1} = X_{i2} = ... = X_{ik}$, we can apply the SUR result and $\hat{\beta}_i = (X_i^T \Sigma_i^{-1} X_i)^{-1} X_i^T \Sigma_i^{-1} Y_i = (X_i^T X_i)^{-1} X_i^T Y_i$ for any R and D.

Y_i can be written as $Y_i = \hat{Y}_i + \hat{\epsilon}_i = X_i\hat{\beta}_i + \hat{\epsilon}_i$, and all information about Y_i contained in $\hat{\beta}_i$, not in $\hat{\epsilon}_i$. From basic theory of linear models, $\hat{\beta}$ is a linear combination of Y_i. As $\hat{\epsilon}_i$ does not contain any information of β_i, $\hat{\beta}$ must be a linear combination of $\hat{\beta}_i$s. Therefore, the minimum variance unbiased estimator (MVUE) of β is given by a linear combination of $\hat{\beta}_i$ with weights inversely proportional to $Var(\hat{\beta}_i)$ derived below.

$$\begin{aligned} Var(\hat{\beta}_i) &= Var((X_i^T X_i)^{-1} X_i^T Y_i) & (1) \\ &= (X_i^T X_i)^{-1} X_i^T var(Y_i) X_i (X_i^T X_i)^{-1} & (2) \\ &= (X_i^T X_i)^{-1} X_i^T (Z_i D Z_i^T + \Sigma_i) X_i (X_i^T X_i)^{-1} & (3) \end{aligned}$$

Because $X_{i1} = ... = X_{ik}$, $Var(\hat{\beta}_i) = V_\beta$, independent of i so that

$\hat{\beta} = (\sum_{i=1}^{n} Var(\hat{\beta}_i)^{-1})^{-1} (\sum_{i=1}^{n} Var(\hat{\beta}_i)^{-1} \hat{\beta}_i) = n^{-1}(\sum_{i=1}^{n} \hat{\beta}_i)$. Since $\hat{\beta}_i$ is identical for combined and separated model in this special case, so is $\hat{\beta}$.

To conclude, estimation of the fixed effects is guaranteed to be more efficient when jointly modeling the measures as compared to separate modeling, except when (1) all the covariance terms across measures are zero in which case the two models are equivalent and (2) $X_{i1} = X_{i2} = ... = X_{ik}$ and $Z_{i1} = Z_{i2} = ... = Z_{ik}$ where Z_{ik} is X_{ik} or a sub-matrix of X_{ik}.

2 Mean squared error and bias-variance decomposition

Since $\hat{\beta}$, $\hat{b}_i$, and $\hat{y}_i$ are functions of the variance estimates, we first obtain the variance estimates by fitting the combined model. We fit a linear mixed effects model with measure-specific smooth function of time as fixed effects plus random intercept and slope. From the model, we obtain fully parameterized covariance estimates of D_C and W_{Ci}, which are assumed to be the true covariances of the underlying population. We obtain D_S and W_{Si} by setting the off-block diagonal elements of D_C and W_{Ci} to zero.

The least squares estimates for fixed effects from the separated and combined models are $\hat{\beta}_S = (X^T W_S X)^{-1} X^T W_S Y$ and $\hat{\beta}_C = (X^T W_C X)^{-1} X^T W_C Y$. Then, as both estimates are unbiased,

$$MSE(\hat{\beta}_S, \beta) = Tr(var(\hat{\beta}_S)) = Tr((X^T W_S X)^{-1} X^T W_S V_C W_S X (X^T W_S X)^{-1})$$
$$MSE(\hat{\beta}_C, \beta) = Tr(var(\hat{\beta}_C)) = Tr((X^T W_C X)^{-1}).$$

We use the conditional expectation of the random effects given the observed data for patient i as our estimates of their random effects. These conditional expectations under the combined and separated models are given by: $\hat{b}_{Si} = D_S Z_i^T W_{Si}(y_i - X_i\hat{\beta}_S)$ and $\hat{b}_{Ci} = D_C Z_i^T W_{C_i}(y_i - X_i\hat{\beta}_C)$.

Following the approach by Laird and Ware (1982) [2], we condition on the true random effect, b_i, then $\hat{b}_{Si}$ and $\hat{b}_{Ci}$ are both biased toward 0. These conditional MSEs are defined as the conditional expected squared difference between the predicted values above and the true value of the random effect. We then obtain the marginal MSE by averaging the conditional values over the distribution of b_i.

For the separated model,

$$E_{b_i}\{MSE(\hat{b}_{Si}, b_i)\} = E_{b_i}\{Tr(var_{\hat{b}_{Si}|b_i}(\hat{b}_{Si}|b_i))\} + E_{b_i}\{||Bias(\hat{b}_{Si})||^2\}$$

$$E_{b_i}\{Tr(var_{y_i|b_i}(\hat{b}_{Si}|b_i))\} = Tr\{D_S Z_i^T(W_{Si} - W_{Si}X_i(X^TW_SX)^{-1}X_i^TW_{Si})\Sigma_{Ci}$$

$$\times(W_{Si} - W_{Si}X_i(X^TW_SX)^{-1}X_i^TW_{Si})Z_iD_S + X_i(X^TW_SX)^{-1}\sum_{n\neq i}^{m} X_n^TW_{Sn}V_{Cn}W_{Sn}X_n\}$$

$$E_{b_i}\{||Bias(\hat{b}_{Si})||^2\} = Tr\{(D_SZ_i^T(W_{Si} - W_{Si}X_i(X^TW_SX)^{-1}X_i^TW_{Si})Z_i - I)D_C$$

$$\times(D_SZ_i^T(W_{Si} - W_{Si}X_i(X^TW_SX)^{-1}X_i^TW_{Si})Z_i - I)^T\}.$$

For the combined model,

$$E_{b_i}\{MSE(\hat{b}_{Ci}, b_i)\} = E_{b_i}\{Tr(var_{\hat{b}_{Ci}|b_i}(\hat{b}_{Ci}|b_i))\} + E_{b_i}\{||Bias(\hat{b}_{Ci})||^2\}$$

$$E_{b_i}\{Tr(var_{y_i|b_i}(\hat{b}_{Ci}|b_i))\} = Tr\{D_C Z_i^T(W_{Ci} - W_{Ci}X_i(X^TW_CX)^{-1}X_i^TW_{Ci})\Sigma_{Ci}$$

$$\times(W_{Ci} - W_{Ci}X_i(X^TW_CX)^{-1}X_i^TW_{Ci})Z_iD_C + X_i(X^TW_CX)^{-1}\sum_{n\neq i}^{m} X_n^TW_{Cn}X_n\}$$

$$E_{b_i}\{||Bias(\hat{b}_{Ci})||^2\} = E_{b_i}\{Tr((E(\hat{b}_{Ci}|b_i) - b_i)(E(\hat{b}_{Ci}|b_i) - b_i)^T)\}$$

$$= Tr\{(D_CZ_i^T(W_{Ci} - W_{Ci}X_i(X^TW_CX)^{-1}X_i^TW_{Ci})Z_i - I)D_C$$

$$\times(D_CZ_i^T(W_{Ci} - W_{Ci}X_i(X^TW_CX)^{-1}X_i^TW_{Ci})Z_i - I)^T\}.$$

The mean squared error (MSE) of an estimator $\theta \in \mathbf{R}^d$ is defined as

$$E(||\hat{\theta} - \theta||^2) = E(\sum_{j=1}^{d}(\hat{\theta}_j - \theta_j)^2) = Tr(Var(\hat{\theta})) + ||Bias(\hat{\theta})||^2$$

where $Bias(\hat{\theta}) = E(\hat{\theta}) - \theta$. Note that $Var(\hat{\theta})$ is the covariance matrix of $\hat{\theta}$ and its trace is $\sum_{j=1}^{d} Var(\hat{\theta}_j)$. Since $MSE(\hat{\theta}, \theta) = \sum_{j=1}^{d} E((\hat{\theta}_j - \theta_j)^2)$, it is sufficient to show $E((\hat{\theta} - \theta)^2) = Var(\hat{\theta}) + Bias^2(\hat{\theta})$ to prove the above result.

$$\begin{aligned}E((\hat{\theta} - \theta)^2) = E((\hat{\theta} - E(\hat{\theta})) + (E(\hat{\theta}) - \theta))^2 = E\{(\hat{\theta} - E(\hat{\theta}))^2 + (E(\hat{\theta}) - \theta)^2\\ + 2(\hat{\theta} - E(\hat{\theta}))(E(\hat{\theta}) - \theta)\}\\ = E(\hat{\theta} - E(\hat{\theta}))^2 + (E(\hat{\theta}) - \theta)^2 + 2(E(\hat{\theta}) - E(\hat{\theta}))(E(\hat{\theta}) - \theta)\}\\ = E(\hat{\theta} - E(\hat{\theta}))^2 + (E(\hat{\theta}) - \theta)^2\\ = Var(\hat{\theta}) + Bias^2(\hat{\theta})\end{aligned}$$

Under our assumptions, $E(Y) = X\beta$ and $var(Y) = V_C$, the fixed effect estimates $\hat{\beta}_S$ and $\hat{\beta}_C$ are unbiased as

$$E(\hat{\beta}_S) = (X^TW_SX)^{-1}X^TW_SE(Y) = \beta$$

$$E(\hat{\beta}_C) = (X^TW_CX)^{-1}X^TW_CE(Y) = \beta$$

and

$$
\begin{aligned}
MSE(\hat{\beta}_S, \beta) &= Tr(var(\hat{\beta}_S)) = Tr((X^T W_S X)^{-1} X^T W_S var(Y) W_S X (X^T W_S X)^{-1}) \\
&= Tr((X^T W_S X)^{-1} X^T W_S V_C W_S X (X^T W_S X)^{-1}) \\
MSE(\hat{\beta}_C, \beta) &= Tr(var(\hat{\beta}_C)) = Tr((X^T W_C X)^{-1})
\end{aligned}
$$

MSE for random effects $\hat{b}_{Si}$ and $\hat{b}_{Ci}$ can be decomposed into variance and bias components. Note that under our assumptions, $var(\epsilon_i) = \Sigma_{Ci}$ and $var(b_i) = D_C$. For the separated model,

$$
E_{b_i}\{MSE(\hat{b}_{Si},\, b_i)\} = E_{b_i}\{Tr(var_{\hat{b}_{Si}|b_i}(\hat{b}_{Si}|b_i))\} + E_{b_i}\{||Bias(\hat{b}_{Si})||^2\}
$$

where the variance component is

$$
E_{b_i}\{Tr(var_{y_i|b_i}(\hat{b}_{Si}|b_i))\} = Tr\{D_S Z_i^T (W_{Si} - W_{Si} X_i (X^T W_S X)^{-1} X_i^T W_{Si}) \Sigma_{Ci}
$$

$$
\times (W_{Si} - W_{Si} X_i (X^T W_S X)^{-1} X_i^T W_{Si}) Z_i D_S + X_i (X^T W_S X)^{-1} \sum_{n \neq i}^{m} X_n^T W_{Sn} V_{Cn} W_{Sn} X_n\}
$$

since $var_{\hat{b}_{Si}|b_i}(\hat{b}_{Si}|b_i) = var_{\hat{b}_{Si}|b_i}(D_S Z_i^T W_{Si}(y_i - X_i \hat{\beta}_S)|b_i)$

$$
\begin{aligned}
&= var_{\hat{b}_{Si}|b_i}(D_S Z_i^T W_{Si}(y_i - X_i (X^T W_S X)^{-1} \sum_{n=1}^{m} X_n^T W_{Sn} y_n)|b_i) \\
&= var_{\hat{b}_{Si}|b_i}(D_S Z_i^T W_{Si}(y_i - X_i (X^T W_S X)^{-1} X_i^T W_{Si} y_i - X_i (X^T W_S X)^{-1} \sum_{n \neq i}^{m} X_n^T W_{Sn} y_n)|b_i) \\
&= var_{\hat{b}_{Si}|b_i}(D_S Z_i^T W_{Si}(I - X_i (X^T W_S X)^{-1} X_i^T W_{Si}) \epsilon_i) \\
&+ var_{\hat{b}_{Si}|b_i}(D_S Z_i^T W_{Si} X_i (X^T W_S X)^{-1} \sum_{n \neq i}^{m} X_n^T W_{Sn} y_n) \\
&= D_S Z_i^T (W_{Si} - W_{Si} X_i (X^T W_S X)^{-1} X_i^T W_{Si}) \Sigma_{Ci} (W_{Si} - W_{Si} X_i (X^T W_S X)^{-1} X_i^T W_{Si}) Z_i D_S \\
&+ D_S Z_i^T W_{Si} X_i (X^T W_S X)^{-1} (\sum_{n \neq i}^{m} X_n^T W_{Sn} V_{Cn} W_{Sn} X_n)(D_S Z_i^T W_{Si} X_i (X^T W_S X)^{-1})^T
\end{aligned}
$$

The bias component is estimated as following

$$
\begin{aligned}
E_{b_i}\{||Bias(\hat{b}_{Si})||^2\} = E_{b_i}\{||(E(\hat{b}_{Si}|b_i) - b_i)||^2\} &= E_{b_i}\{Tr((E(\hat{b}_{Si}|b_i) - b_i)(E(\hat{b}_{Si}|b_i) - b_i)^T)\} \\
&= Tr\{(D_S Z_i^T (W_{Si} - W_{Si} X_i (X^T W_S X)^{-1} X_i^T W_{Si}) Z_i - I) E_{b_i}(b_i b_i^T) \\
&\times (D_S Z_i^T (W_{Si} - W_{Si} X_i (X^T W_S X)^{-1} X_i^T W_{Si}) Z_i - I)^T\} \\
&= Tr\{(D_S Z_i^T (W_{Si} - W_{Si} X_i (X^T W_S X)^{-1} X_i^T W_{Si}) Z_i - I) D_C \\
&\times (D_S Z_i^T (W_{Si} - W_{Si} X_i (X^T W_S X)^{-1} X_i^T W_{Si}) Z_i - I)^T\}
\end{aligned}
$$

since

$$\begin{aligned}
E_{\hat{b}_{Si}|b_i}(\hat{b}_{Si}|b_i) &= E_{\hat{b}_{Si}|b_i}(D_S Z_i^T W_{Si}(y_i - X_i\hat{\beta}_S)|b_i) \\
&= E_{\hat{b}_{Si}|b_i}(D_S Z_i^T W_{Si}(y_i - X_i(X^T W_S X)^{-1}\sum_{n=1}^{m} X_n^T W_{Sn} y_n)|b_i) \\
&= E_{\hat{b}_{Si}|b_i}(D_S Z_i^T W_{Si}\{y_i - X_i(X^T W_S X)^{-1} X_i^T W_{Si} y_i \\
&- X_i(X^T W_S X)^{-1}\sum_{n\neq i}^{m} X_n^T W_{Sn} y_n\}|b_i) \\
&= D_S Z_i^T W_{Si}\{(I - X_i(X^T W_S X)^{-1} X_i^T W_{Si})(X_i\beta + Z_i b_i) \\
&- X_i(X^T W_S X)^{-1}\sum_{n\neq i}^{m} X_n^T W_{Sn} X_n\beta\} \\
&= D_S Z_i^T W_{Si}\{(I - X_i(X^T W_S X)^{-1} X_i^T W_{Si}) Z_i b_i \\
&+ (I - X_i(X^T W_S X)^{-1} X_i^T W_{Si}) X_i\beta - X_i(X^T W_S X)^{-1}\sum_{n\neq i}^{m} X_n^T W_{Sn} X_n\beta\} \\
&= D_S Z_i^T W_{Si}\{(I - X_i(X^T W_S X)^{-1} X_i^T W_{Si}) Z_i b_i \\
&+ X_i\beta - X_i(X^T W_S X)^{-1}\sum_{n=1}^{m} X_n^T W_{Sn} X_n\beta\} \\
&= D_S Z_i^T W_{Si}\{(I - X_i(X^T W_S X)^{-1} X_i^T W_{Si}) Z_i b_i\}.
\end{aligned}$$

Similarly, for the combined model

$$E_{b_i}\{MSE(\hat{b}_{Ci}, b_i)\} = E_{b_i}\{Tr(var_{\hat{b}_{Ci}|b_i}(\hat{b}_{Ci}|b_i))\} + E_{b_i}\{||Bias(\hat{b}_{Ci})||^2\}$$

where the variance component is

$$\begin{aligned}
E_{b_i}\{Tr(var_{y_i|b_i}(\hat{b}_{Ci}|b_i))\} &= Tr\{D_C Z_i^T (W_{Ci} - W_{Ci} X_i (X^T W_C X)^{-1} X_i^T W_{Ci})\Sigma_{Ci} \\
\times(W_{Ci} - W_{Ci} X_i (X^T W_C X)^{-1} X_i^T W_{Ci}) Z_i D_C &+ X_i (X^T W_C X)^{-1}\sum_{n\neq i}^{m} X_n^T W_{Cn} X_n\}
\end{aligned}$$

since $var_{\hat{b}_{Ci}|b_i}(\hat{b}_{Ci}|b_i) = var_{\hat{b}_{Ci}|b_i}(D_C Z_i^T W_{Ci}(y_i - X_i\hat{\beta}_C)|b_i)$

$$\begin{aligned}
&= var_{\hat{b}_{Ci}|b_i}(D_C Z_i^T W_{Ci}(I - X_i(X^T W_C X)^{-1} X_i^T W_{Ci})\epsilon_i) \\
&+ var_{\hat{b}_{Ci}|b_i}(D_C Z_i^T W_{Ci} X_i (X^T W_C X)^{-1}\sum_{n\neq i}^{m} X_n^T W_{Cn} y_n) \\
&= D_C Z_i^T (W_{Ci} - W_{Ci} X_i (X^T W_C X)^{-1} X_i^T W_{Ci})\Sigma_{Ci}(W_{Ci} - W_{Ci} X_i (X^T W_C X)^{-1} X_i^T W_{Ci}) Z_i D_C \\
&+ D_C Z_i^T W_{Ci} X_i (X^T W_C X)^{-1}\sum_{n\neq i}^{m} X_n^T W_{Cn} X_n (D_C Z_i^T W_{Ci} X_i (X^T W_C X)^{-1})^T
\end{aligned}$$

The bias component is

$$\begin{aligned}
E_{b_i}\{||Bias(\hat{b}_{Ci})||^2\} &= E_{b_i}\{Tr((E(\hat{b}_{Ci}|b_i) - b_i)(E(\hat{b}_{Ci}|b_i) - b_i)^T)\} \\
&= Tr\{(D_C Z_i^T (W_{Ci} - W_{Ci} X_i (X^T W_C X)^{-1} X_i^T W_{Ci}) Z_i - I) D_C \\
&\times(D_C Z_i^T (W_{Ci} - W_{Ci} X_i (X^T W_C X)^{-1} X_i^T W_{Ci}) Z_i - I)^T\}
\end{aligned}$$

since $E_{\hat{b}_{Ci}|b_i}(\hat{b}_{Ci}|b_i) = E_{\hat{b}_{Ci}|b_i}(D_C Z_i^T W_{Ci}(y_i - X_i\hat{\beta}_C)|b_i)$

$= D_C Z_i^T W_{Ci}\{(I - X_i(X^T W_C X)^{-1} X_i^T W_{Ci}) Z_i b_i\}$.

Lastly, MSE for $\hat{y}_{Si} = X_i\hat{\beta}_S + Z_i\hat{b}_i = X_i\hat{\beta}_S + Z_i D_S Z_i^T W_{Si}(y_i - X_i\hat{\beta}_S)$ can be written as

$$E_{b_i}\{MSE(\hat{y}_{Si},\, E(\hat{y}_i|b_i))\} = E_{b_i}\{Tr(var_{\hat{y}_{Si}|b_i}(\hat{y}_{Si}|b_i))\} + E_{b_i}\{||Bias(\hat{y}_{Si})||^2\}$$

$E_{\hat{y}_{Si}|b_i}(\hat{y}_{Si}|b_i)$ and $var_{\hat{y}_{Si}|b_i}(\hat{y}_{Si}|b_i)$ is derived where $M_{Si} = (X_i - Z_i D_S Z_i^T W_{Si} X_i)(X^T W_S X)^{-1}$.

$$\begin{aligned}
E_{\hat{y}_{Si}|b_i}(\hat{y}_{Si}|b_i) &= E_{\hat{y}_{Si}|b_i}(X_i\hat{\beta}_S + Z_i D_S Z_i^T W_{Si}(y_i - X_i\hat{\beta}_S)) \\
&= E_{\hat{y}_{Si}|b_i}\{M_{Si}\sum_{n=1}^{m} X_n^T W_{Sn} y_n + Z_i D_S Z_i^T W_{Si} y_i\} \\
&= E_{\hat{y}_{Si}|b_i}\{M_{Si} X_i^T W_{Si} y_i + Z_i D_S Z_i^T W_{Si} y_i + M_i \sum_{n\neq i}^{m} X_n^T W_{Sn} y_n\} \\
&= M_{Si} X_i^T W_{Si}(X_i\beta + Z_i b_i) + Z_i D_S Z_i^T W_{Si}(X_i\beta + Z_i b_i) + M_{Si}\sum_{n\neq i}^{m} X_n^T W_{Sn} X_n\beta \\
&= X_i\beta + \{M_{Si} X_i^T + Z_i D_S Z_i^T\} W_{Si} Z_i b_i
\end{aligned}$$

$$\begin{aligned}
var_{\hat{y}_{Si}|b_i}(\hat{y}_{Si}|b_i) &= var_{\hat{y}_{Si}|b_i}\{M_{Si} X_i^T W_{Si} y_i + Z_i D_S Z_i^T W_{Si} y_i + M_{Si}\sum_{n\neq i}^{m} X_n^T W_{Sn} y_n\} \\
&= (M_{Si} X_i^T + Z_i D_S Z_i^T) W_{Si}\Sigma_{Ci} W_{Si}(M_{Si} X_i^T + Z_i D_S Z_i^T)^T \\
&+ M_{Si}(\sum_{n\neq i}^{m} X_n^T W_{Sn} V_{Cn} W_{Sn} X_n) M_{Si}^T
\end{aligned}$$

Hence,

$$\begin{aligned}
E_{b_i}\{Tr(var_{\hat{y}_{Si}|b_i}(\hat{y}_{Si}|b_i))\} &= Tr((M_{Si} X_i^T + Z_i D_S Z_i^T) W_{Si}\Sigma_{Ci} W_{Si}(M_{Si} X_i^T + Z_i D_S Z_i^T)^T \\
&+ M_{Si}(\sum_{n\neq i}^{m} X_n^T W_{Sn} V_{Cn} W_{Sn} X_n) M_{Si}^T)
\end{aligned}$$

and

$$\begin{aligned}
E_{b_i}\{||Bias(\hat{y}_{Si})||^2\} &= E_{b_i}\{Tr((E(\hat{y}_{Si}|b_i) - (X_i\beta + Z_i b_i))(E(\hat{y}_{Si}|b_i) - (X_i\beta + Z_i b_i))^T)\} \\
&= Tr(E_{b_i}\{(\{M_{Si} X_i^T + Z_i D_S Z_i^T\} W_{Si} - I) Z_i b_i b_i^T Z_i(\{M_{Si} X_i^T + Z_i D_S Z_i^T\} W_{Si} - I)^T\}) \\
&= Tr((\{M_{Si} X_i^T + Z_i D_S Z_i^T\} W_{Si} - I) Z_i D_{Ci} Z_i(\{M_{Si} X_i^T + Z_i D_S Z_i^T\} W_{Si} - I)^T)
\end{aligned}$$

MSE for $\hat{y}_{Ci} = X_i\hat{\beta}_C + Z_i D_C Z_i^T W_{Ci}(y_i - X_i\hat{\beta}_C)$ is

$$E_{b_i}\{MSE(\hat{y}_{Ci},\, E(\hat{y}_i|b_i))\} = E_{b_i}\{Tr(var_{\hat{y}_{Ci}|b_i}(\hat{y}_{Ci}|b_i))\} + E_{b_i}\{||Bias(\hat{y}_{Ci})||^2\}$$

Letting $M_{Ci} = (X_i - Z_i D_C Z_i^T W_{Ci} X_i)(X^T W_C X)^{-1}$,

$$\begin{aligned}
E_{\hat{y}_{Si}|b_i}(\hat{y}_{Ci}|b_i) &= E_{\hat{y}_{Ci}|b_i}(X_i\hat{\beta}_C + Z_i D_C Z_i^T W_{Ci}(y_i - X_i\hat{\beta}_C)) \\
&= E_{\hat{y}_{Ci}|b_i}\{M_{Si}\sum_{n=1}^{m} X_n^T W_{Cn} y_n + Z_i D_C Z_i^T W_{Ci} y_i\} \\
&= E_{\hat{y}_{Ci}|b_i}\{M_{Ci} X_i^T W_{Ci} y_i + Z_i D_C Z_i^T W_{Ci} y_i + M_i \sum_{n\neq i}^{m} X_n^T W_{Cn} y_n\} \\
&= M_{Ci} X_i^T W_{Ci}(X_i\beta + Z_i b_i) + Z_i D_C Z_i^T W_{Ci}(X_i\beta + Z_i b_i) + M_{Ci}\sum_{n\neq i}^{m} X_n^T W_{Cn} X_n\beta \\
&= X_i\beta + \{M_{Ci} X_i^T + Z_i D_C Z_i^T\} W_{Ci} Z_i b_i
\end{aligned}$$

$$var_{\hat{y}_{Ci}|b_i}(\hat{y}_{Ci}|b_i) = var_{\hat{y}_{Ci}|b_i}\{M_{Ci}X_i^T W_{Ci} y_i + Z_i D_C Z_i^T W_{Ci} y_i + M_{Ci}\sum_{n\neq i}^{m} X_n^T W_{Cn} y_n\}$$
$$= (M_{Ci}X_i^T + Z_i D_C Z_i^T) W_{Ci}\Sigma_{Ci} W_{Ci}(M_{Ci}X_i^T + Z_i D_C Z_i^T)^T$$
$$+ M_{Ci}(\sum_{n\neq i}^{m} X_n^T W_{Cn} X_n) M_{Ci}^T$$

Hence,

$$E_{b_i}\{Tr(var_{\hat{y}_{Ci}|b_i}(\hat{y}_{Ci}|b_i))\} = Tr((M_{Ci}X_i^T + Z_i D_C Z_i^T) W_{Ci}\Sigma_{Ci} W_{Ci}(M_{Ci}X_i^T + Z_i D_C Z_i^T)^T)$$
$$+ M_{Ci}(\sum_{n\neq i}^{m} X_n^T W_{Cn} X_n) M_{Ci}^T$$

and

$$E_{b_i}\{||Bias(\hat{y}_{Ci})||^2\} = E_{b_i}\{Tr((E(\hat{y}_{Ci}|b_i) - (X_i\beta + Z_i b_i))(E(\hat{y}_{Ci}|b_i) - (X_i\beta + Z_i b_i))^T)\}$$
$$= Tr(E_{b_i}\{(\{M_{Ci}X_i^T + Z_i D_C Z_i^T\} W_{Ci} - I) Z_i b_i b_i^T Z_i (\{M_{Ci}X_i^T + Z_i D_C Z_i^T\} W_{Ci} - I)^T\})$$
$$= Tr((\{M_{Ci}X_i^T + Z_i D_C Z_i^T\} W_{Ci} - I) Z_i D_{Ci} Z_i (\{M_{Ci}X_i^T + Z_i D_C Z_i^T\} W_{Ci} - I)^T)$$

3 Mean squared error and bias-variance decomposition of random effect estimates with known population parameters

With known β, the bias and variance components are calculated as following.

For the seperated model,

$$var_{\hat{b_{Si}}|b_i}(\hat{b_{Si}}|b_i) = var(D_S Z_i^T W_{S_i}(y_i - X_i\beta)|b_i)$$
$$= var(D_S Z_i^T W_{S_i}(Z_i b_i + \epsilon_i)|b_i) = var(D_S Z_i^T W_{S_i}\epsilon_i) = D_S Z_i^T W_{S_i}\Sigma_{C_i} W_{S_i} Z_i D_S$$
$$E_{b_i}\{Tr(var_{\hat{b_{Si}}|b_i}(\hat{b_{Si}}|b_i))\} = Tr\{D_S Z_i^T W_{S_i}\Sigma_{C_i} W_{S_i} Z_i D_S\}$$

$$E(\hat{b_{Si}}|b_i) = E(D_S Z_i^T W_{S_i}(Z_i b_i + \epsilon_i)|b_i) = D_S Z_i^T W_{S_i} Z_i b_i$$
$$E\{||Bias(\hat{b}_{S_i})||^2\} = E\{||(E(\hat{b_{S_i}}|b_i) - b_i)(E(\hat{b_{S_i}}|b_i) - b_i)^T)||\}$$
$$= (D_S Z_i^T W_{S_i} Z_i - I) E(b_i b_i^T)(D_S Z_i^T W_{S_i} Z_i - I)^T$$
$$= (D_S Z_i^T W_{S_i} Z_i - I) D_C (D_S Z_i^T W_{S_i} Z_i - I)^T$$

Similarly for the combined model,

$$var_{\hat{b_{Ci}}|b_i}(\hat{b_{Ci}}|b_i) = var(D_C Z_i^T W_{C_i}(y_i - X_i\beta)|b_i)$$
$$= var(D_C Z_i^T W_{C_i}(Z_i b_i + \epsilon_i)|b_i) = var(D_C Z_i^T W_{C_i}\epsilon_i) = D_C Z_i^T W_{C_i}\Sigma_{C_i} W_{C_i} Z_i D_C$$
$$E_{b_i}\{Tr(var_{\hat{b_{Ci}}|b_i}(\hat{b_{Ci}}|b_i))\} = Tr\{D_C Z_i^T W_{C_i}\Sigma_{C_i} W_{C_i} Z_i D_C\}$$
$$E\{||Bias(\hat{b}_{C_i})||^2\} = (D_C Z_i^T W_{C_i} Z_i - I) D_C (D_C Z_i^T W_{C_i} Z_i - I)^T$$

4 Efficiency gains for the random effects in the case of drop-out missing pattern

We show efficiency gains for the random effects b_{i2} for the second measure Y_{i2} in the case of drop-out missing pattern representing right truncated data. S1 Fig plots ratios of MSEs of the combined model to separated model for combinations of ρ_b and ρ_r and S2 Fig shows the ratios for $n_{i1} = 6$ case under different scenarios. The gains are slightly greater by fitting the combined model in drop-out missing pattern compared to the randomly missing case, but the findings are qualitatively same.

Fig 1. MSE Ratio of b_{i2} by varying p_{miss}, ρ_b and ρ_r under scenarios A, B, and C when $n_{i1} = 6, \rho_k = 0$ for drop-out missing case. Cells representing unlikely combinations of ρ_b and ρ_r are colored in grey.

Fig 2. MSE Ratio of b_{i2} evaluated at $p_{miss} = 0\%, 33\%, 66\%$, $n_{i1} = N = 6, 9, 15$, $\rho_r = -0.9, -0.7, ..., 0.7, 0.9$, $\rho_b = -0.9, -0.7, ..., 0.7, 0.9$, $\rho_k = -0.5, 0, 0.5$ under cases A, B, and C for drop-out missing pattern. Only the "likely combinations" of ρ_b and ρ_r illustrated are used to create boxplots. Different combinations of ρ_k^1 and ρ_k^2 yield negligible difference in outcome, so we only present the case of common ρ_k that takes three values.

References

1. Zellner, A. An Efficient Method of Estimating Seemingly Unrelated Regressions and Tests for Aggregation Bias. *Journal Of The American Statistical Association.* **57**, 348-368 (1962)

2. Laird, N. & Ware, J. Random-effects models for longitudinal data.. *Biometrics.* **38** pp. 963-974 (1982)
