# Supplementary figures and images for: Separated or joint models of repeated multivariate data to estimate individuals’ disease trajectories with application to scleroderma
Source: PLoS One. 2025 Apr 21;20(4):e0320414. doi: 10.1371/journal.pone.0320414 (PMC12011310; doi:10.1371/journal.pone.0320414)

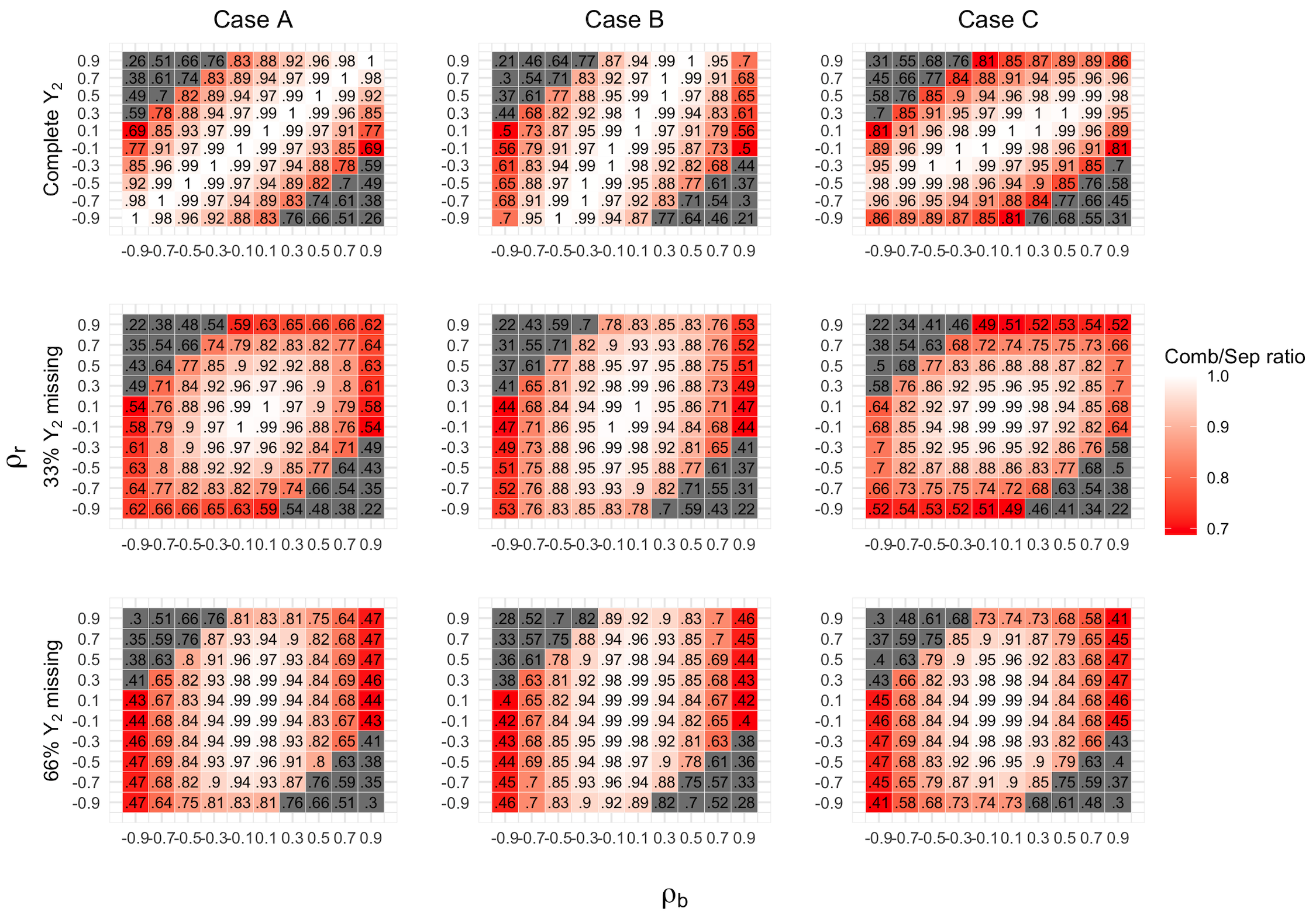

Supplement: S1 Fig — (TIF) [file pone.0320414.s002.tif]

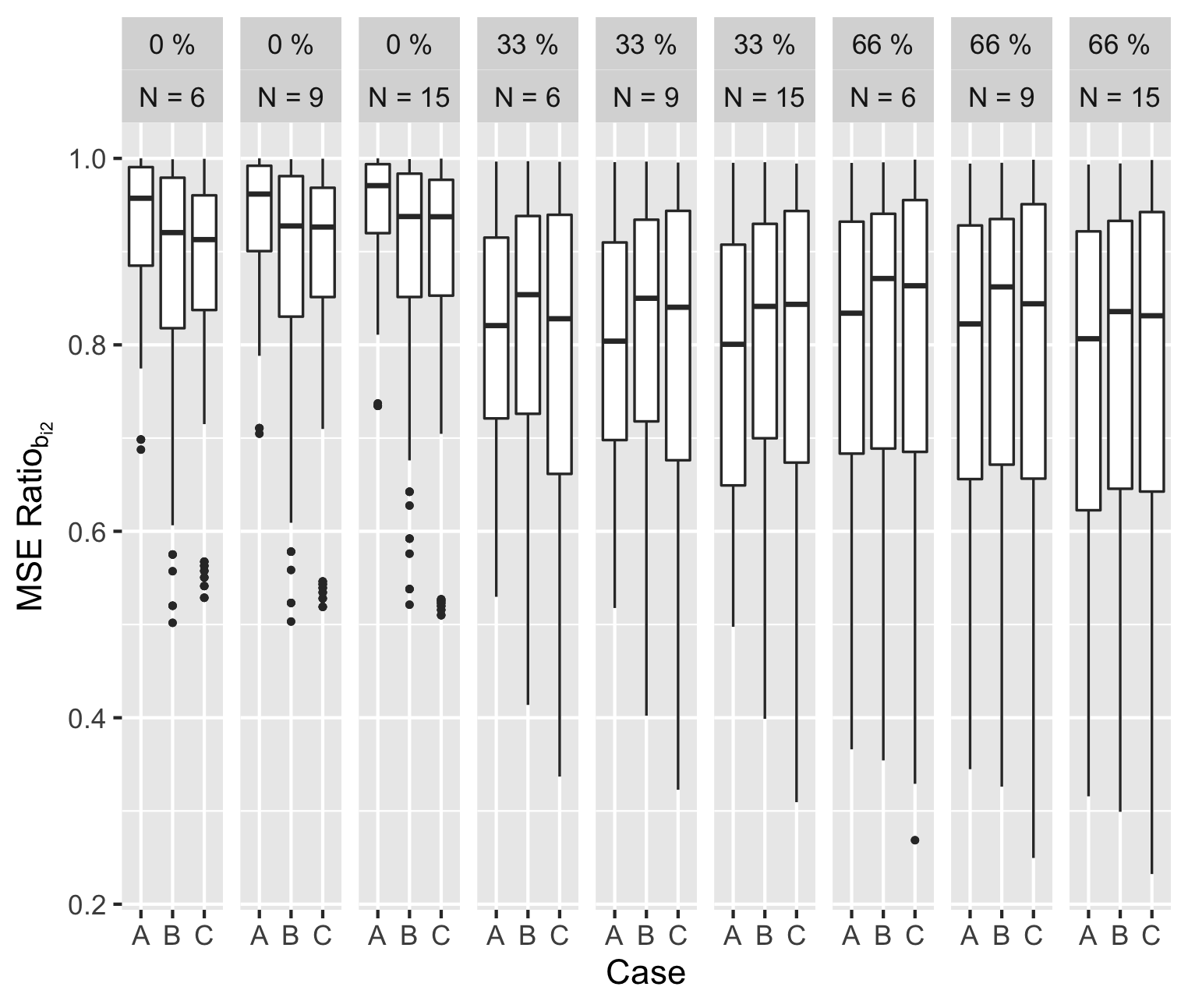

Supplement: S2 Fig — (TIF) [file pone.0320414.s003.tif]
